# A cohort study of the association between maternal serum Inhibin-A and adverse pregnancy outcomes: a population-based study

**DOI:** 10.1186/s12884-019-2266-y

**Published:** 2019-04-11

**Authors:** Wannaporn Singnoi, Chanane Wanapirak, Ratanaporn Sekararithi, Theera Tongsong

**Affiliations:** Department of Obstetrics and Gynecology, Faculty of Medicine, Chiang Mai University, Chiang Mai, 50200 Thailand

**Keywords:** Fetal growth restriction, Inhibin-a, Preeclampsia, Preterm birth, Serum marker screening

## Abstract

**Background:**

To compare the rates of adverse pregnancy outcomes between women with normal and abnormal inhibin-A levels.

**Methods:**

Based on a prospective database of Down syndrome screening program, the consecutive records were comprehensively reviewed. Pregnancies were classified into three groups: normal, high (> 2 MoM) and low (< 0.5 MoM) inhibin-A levels. The pregnancies with medical diseases, chromosome abnormalities and fetal anomalies were excluded. The primary outcomes were the rates of preterm birth, preeclampsia, and fetal growth restriction (FGR).

**Results:**

Of 6679 recruited pregnancies, 5080 met the inclusion criteria, including 4600, 205 and 275 pregnancies in the group of normal, high, and low inhibin-A levels respectively. The rates of preterm birth, preeclampsia and FGR were significantly higher in the group of high levels; (RR, 1.51, 95%CI: 1.01–2.26; 3.47, 95% CI: 2.13–5.65; 3.04, 95% CI: 1.99–4.65 respectively), whereas the rates of other adverse outcomes were comparable. However, the rate of spontaneous preterm birth among women with high inhibin-A was not significantly increased. Based on multivariate analysis, the preterm birth rate was not significantly associated with inhibin-A levels, but it was rather a consequence of preeclampsia and FGR. Low levels of serum inhibin-A were not significantly associated with any adverse outcomes.

**Conclusions:**

High levels of maternal serum inhibin-A in the second trimester are significantly associated with abnormal placentation, which increases the risk of preeclampsia and FGR with a consequence of indicated preterm birth but not a risk of spontaneous preterm birth. In contrast, low inhibin-A levels were not associated with any common adverse pregnancy outcomes.

## Background

The quad test or quadruple test is the most commonly used serum screening test for fetal Down syndrome, consisting of alpha-fetoprotein (AFP), human chorionic gonadotrophin (hCG), unconjugated estriol (uE3) and inhibin-A. According to a systematic review in 2012 [1], quad test has a detection rate of 80% at a false positive rate of 5%. The quad test was used for the first time in 1996 [2], and currently, it has been used worldwide. With extensive experience, much evidence of the association between abnormal serum biomarker levels and pregnancy outcomes has also been accumulated. Several studies indicate that abnormal biomarker levels may be associated with poor pregnancy outcomes, in particular preterm birth, intrauterine growth restriction and preeclampsia. Thus, we can probably use such serum markers in the second trimester to predict outcomes in late gestation.

Also preterm birth and preeclampsia are serious obstetric problems experienced worldwide. Several attempts have been made to prevent preterm birth and preeclampsia. For example, progesterone administration is recommended for preterm birth prevention in women with higher risk of spontaneous preterm birth [3], and aspirin is indicated to prevent preeclampsia in cases of high risk [4]. Accordingly, risk identification is essential for patient selection to achieve proper antenatal care. As mentioned above, serum biomarkers may be used as a predictor of adverse pregnancy outcomes; they could be useful when combined with other risk factors.

Currently, we have implemented a screening program for fetal Down syndrome with serum markers in second-trimester pregnant women, as a pilot-study project in Thailand. All the participants underwent quad test free of charge, supported by the government. Under the project, baseline data, laboratory results and obstetric outcomes were followed-up and prospectively recorded. Accordingly, we could take advantage of such screening to study the association between various serum biomarkers and adverse pregnancy outcomes. Several studies have been published on the association between unexplained abnormal serum biomarker levels, including the first trimester serum markers (beta-hCG and PAPP-A) and second trimester triple serum markers (AFP, beta-hCG and uE3), and adverse pregnancy outcomes, especially preeclampsia, fetal growth restriction and preterm births [5–8]. Nevertheless, studies on the association between inhibin-A as a component of quad test and pregnancy outcomes are limited [9–11], especially in the Asian population. Additionally, the results from previous studies are inconsistent [1, 11]. Abnormal levels of inhibin-A may be helpful in predicting various adverse outcomes, especially when combined with other risk factors. We hypothesize that abnormal levels of inhibin-A could possibly be reflective of the abnormality of the feto-placental unit, leading to adverse pregnancy outcomes. Therefore, we conducted this study to compare the rates of adverse pregnancy outcomes, in particular preeclampsia, fetal growth restriction, preterm delivery and low birthweight, between women with normal and abnormal inhibin-A levels.

## Methods

A cohort study, as a secondary analysis, was conducted with ethical approval by the Institute Review Board, based on a prospective database of Down syndrome screening program by quad test. On the database development, the women were recruited with written informed consent at Chiang Mai University hospital and its network of hospitals in the northern part of Thailand. The consecutive records between October 2016 and March 2018 were assessed and comprehensively reviewed. The inclusion criteria were as follows: 1) singleton pregnancy and 2) gestational age of 15–20 weeks, based on ultrasound biometry of crown-rump length in the first trimester or biparietal diameter in the first half of pregnancy. The exclusion criteria were as follows: 1) fetal structural or chromosomal abnormalities, 2) pregnancy with medical disorders such as heart disease, pre-gestational diabetes mellitus, chronic hypertension, etc., and 3) loss to follow-up, unknown pregnancy outcomes or unavailable data. Pregnancies were categorized into three groups, including the group of normal inhibin-A levels, high levels (> 2 MoM) and low levels (< 0.5 MoM). The primary outcomes were the rates of preterm birth, preeclampsia, and fetal growth restriction (FGR). Additionally, route of delivery, low APGAR scores as well as antepartum and postpartum hemorrhage were also assessed as secondary outcomes.

The baseline data of the participants were prospectively evaluated and recorded in the research record form at the time of maternal blood sampling and stored in the computerized database at our research center, Maharaj Nakorn Chiang Mai Hospital and its network of hospitals. The baseline characteristics were as follows: maternal age, parity, ethnicity, height, body weight, body mass index (BMI), gestational age, medical diseases, familial diseases, education, occupations, and smoking habit. The maternal blood samples were then sent to the laboratory and were centrifuged for separation of blood components. The bio-assay procedures of inhibin-A were performed by the same experienced technicians at the research center. The bio-assays for inhibin-A were carried out in batches to get rid of inter-assay variations. The bio-assays were run using DELFIA® Xpress system (Perkin Elmer, Waltham, MA). The laboratory quality control was regularly validated by third-party experts under the primary project of the National Research University and Thailand’s Office of the Higher Education Commission. The measured levels of maternal serum inhibin-A were then automatically converted to MoM (multiple of medians), using the built-in western reference ranges with ethnic correction; the MoMs were also corrected for maternal weight, smoking and diabetes.

The enrolled participants who met the inclusion criteria were divided into three subgroups as follows: [1] normal inhibin-A levels (0.5–2.0 MoM), [2] high inhibin-A levels (more than 2.0 MoM), and [3] low inhibin-A levels (less than 0.5 MoM). All pregnant women were followed up until pregnancy completion to assess the obstetric outcomes. The obstetric outcomes and neonatal outcomes were evaluated by our research teams and the neonatologists in the network of hospitals. The primary outcomes that were analyzed after excluding fetal anomaly or chromosome abnormality, abortion and maternal medical conditions were the prevalence of preterm birth, FGR, and preeclampsia. The secondary outcomes included route of delivery, LBW (low birth weight), low Apgar scores at 5 min, antepartum and postpartum hemorrhage.

Definitions of obstetric outcomes were as follows: 1) preterm delivery: giving birth before 37 complete weeks of gestation, including spontaneous and indicated preterm birth (intentional preterm such as induction of labor in cases of severe preterm preeclampsia), 2) FGR: fetuses with birth weight lower than the 10th percentile, using the fetal growth rate graph of Thai reference ranges, 3) Preeclampsia: systolic BP > 140 mmHg or diastolic BP > 90 mmHg together with proteinuria 1+ or more on urinary dipstick or urine protein creatinine ratio more than 0.3 or 24-h urinary protein greater than 300 mg after 20 gestational weeks, 4) LBW (low birth weight): birth weight less than 2500 g, 5) low APGAR scores: the scores of less than 7 at 5 min, 6) antepartum hemorrhage: uterine bleeding after 20 gestational weeks, and [7] postpartum hemorrhage: bleeding after delivery more than 500 ml in vaginal delivery and 1000 ml in cesarean delivery.

### Statistical analysis

The baseline characteristics between the normal and abnormal groups were compared by Student’s T-test or Mann-Whitney-U for continuous variables and Chi-square test for categorical data. The percentages of preterm birth, preeclampsia, FGR, low birth weight, low APGAR scores, antepartum hemorrhage and postpartum hemorrhage were compared between the groups of abnormal (high and low) inhibin-A concentrations and the group of normal concentrations, using Chi square as well as relative risks with 95% CI. Binary logistic regression analysis was performed to adjust the confounders of the main outcomes. *P*-value < 0.05 was considered statistically significant. The statistical analysis was done with SPSS (IBM Corp. Released 2012; IBM SPSS Statistics for Windows, Version 21.0. Armonk, NY).

## Results

**Fig. 1:**
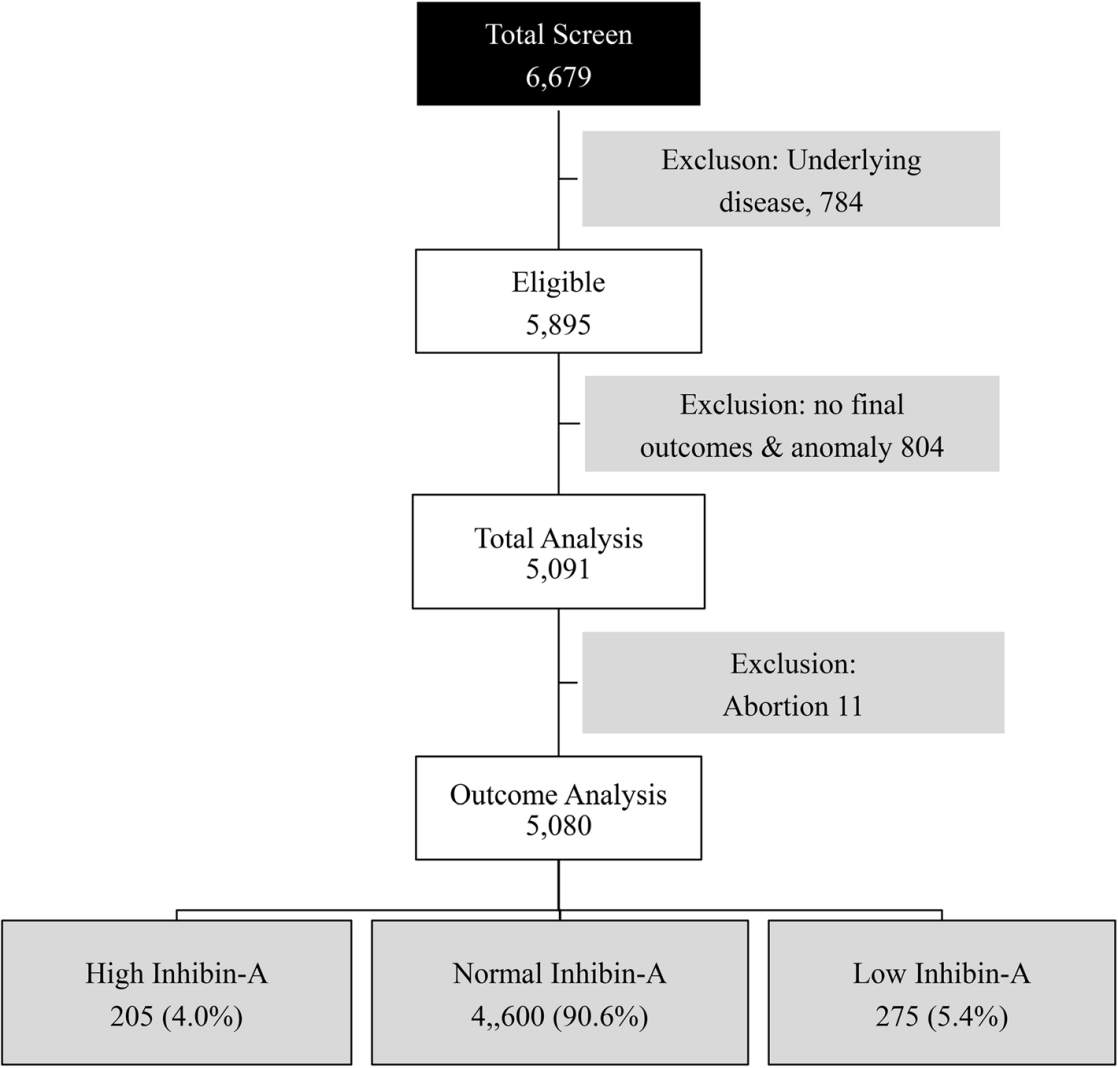
Flowchart of the participant recruitment

During the study period, 6679 pregnancies undergoing fetal Down syndrome screening with quad test were recruited. Of this number, 1599 were excluded because of medical conditions (804), such as chronic hypertension, diabetes mellitus, heart disease etc., unavailable pregnancy outcomes, fetal anomalies or chromosome abnormalities (804), and abortion before 20 weeks [12], as presented in Fig. 1. The remaining 5080 pregnancies met the inclusion criteria and were available for analysis, including 4600 (90.6%), 205 (4.0%) and 275 (5.4%) pregnancies in the group of normal levels, high levels, and low levels of serum inhibin-A, respectively. All the baseline characteristics of the three groups were comparable, as presented in Table 1.

**Table 1:** Baseline characteristics of pregnancy between the group of high level, low level of inhibin A and normal group

	Low IHA (*N* = 275)	Normal IHA (*N* = 4600)	High IHA (*N* = 205)	*P* value
Age (year) (mean ± SD)	27.20 ± 5.03	28.32 ± 14.48	29.43 ± 5.84	0.212
Body Weight (kg) (mean ± SD)	56.38 ± 12.28	57.38 ± 11.25	57.84 ± 11.67	0.300
Body mass index (kg/m^2^) (mean ± SD)	22.77 ± 4.43	23.21 ± 4.28	23.46 ± 4.32	0.170
Parity				0.450
Nulliparity (n) (%)	108 (39.3%)	1978 (43.0%)	90 (43.9%)	
Multiparity (n) (%)	167 (60.7%)	2618 (57.0%)	115 (56.1%)	
Socioeconomic status^a^				0.470
Low (n) (%)	183 (66.5%)	3222 (70.0%)	143 (69.8%)	
High (n) (%)	92 (33.5%)	1378 (30.0%)	62 (30.2%)	
Smoking				0.587
Non-smoker (n) (%)	270 (99.6%)	4539 (99.8%)	195 (100%)	
Smoker (n) (%)	1 (0.4%)	7 (0.2%)	0 (0%)	
Gestational age at screening (mean ± SD)	15.38 ± 1.53	15.56 ± 2.08	15.44 ± 2.27	0.306
Gestational age at delivery (mean ± SD)	38.36 ± 1.34	38.30 ± 1.86	38.09 ± 2.34	0.243

^a^Based on their occupations; *IHA* Inhibin-A

### Comparison of the adverse pregnancy outcomes between the groups of high and normal inhibin-a levels

The analysis revealed that the rates of preterm birth, preeclampsia, fetal growth restriction, and low birth weight were significantly higher in the group of high levels than those in the normal group; (12.2% vs 8.3%, *p*-value 0.049, 7.3% vs 2.0%, *p*-value < 0.001; 10.2% vs 3.3%, *p*-value < 0.001; and 15.1% vs 9.5%, p-value 0.008 respectively), while the percentage of other adverse obstetric outcomes, including low APGAR scores, antepartum hemorrhage, postpartum hemorrhage and route of delivery were not significantly different between the two groups, as presented in Table 2. However, after excluding indicated preterm birth such as preeclampsia, fetal growth restriction with non-reassuring fetal heart rate, antepartum hemorrhage, etc. the rate of spontaneous preterm birth among the high inhibin-A group was not significantly different from the rate in the normal inhibin-A group (5.4% vs 7.0%, *p*-value 0.356). Likewise, after adjusting for the potential confounding factors by logistic regression analysis, preterm birth was not significantly associated with high inhibin-A (*p*-value 0.475) but significantly related to preeclampsia (*p*-value < 0.001) and advanced maternal age (*p*-value 0.030), as presented in Table 3. In the analysis of adjusted odd ratios for risk factors of preeclampsia, high inhibin-A was still a significant risk (odd ratio of 3.77, 95%CI: 2.15–6.63; *p*-value < 0.001), as shown in Table 3.

**Table 2:** Comparison of adverse pregnancy outcomes between the group of high level and normal group of inhibin-A level

	Normal IHA (*N* = 4600)	High IHA (> 2.0 MoM) (*N* = 205)	Relative Risk (95% CI)	*P* value	Low IHA (> 2.0 MoM) (*N* = 275)	Relative Risk (95% CI)	*P* value
Preterm birth	381 (8.3%)	25 (12.2%)	1.51 (1.01–2.26)	0.049	19 (6.9%)	0.83 (0.53–1.31)	0.420
Spontaneous preterm birth	324 (7.0%)	11 (5.4%)	0.76 (0.42–1.37)	0.356	15 (5.5%)	0.77 (0.46–1.28)	0.314
Preeclampsia	92 (2.0%)	15 (7.3%)	3.47 (2.13–5.65)	< 0.001	7 (2.5%)	1.26 (0.61–2.60)	0.533
FGR	153 (3.3%)	21 (10.2%)	3.04 (1. 99–4.65)	< 0.001	12 (4.4%)	1.30 (0.75–2.27)	0.355
Low birth weight	437 (9.5%)	31 (15.1%)	1.65 (1.14–2.39)	0.008	24 (8.7%)	0.92 (0.61–1.38)	0.671
Antepartum hemorrhage	25 (0.5%)	3 (1.5%)	2.53 (0.86–7.44)	0.091	1 (0.4%)	0.68 (0.10–4.66)	0.690
Postpartum hemorrhage	78 (1.7%)	5 (2.4%)	1.42 (0.60–3.35)	0.427	1 (0.4%)	0.22 (0.03–1.55)	0.089
Low APGAR scores	36 (0.8%)	2 (1.0%)	1.01 (0.94–1.09)	0.760	3 (1.1%)	1.02 (0.93–1.12)	0.577
Route of delivery				0.401			0.175
Normal delivery	2659 (57.9%)	116 (56.6%)			174 (63.3%)		
Cesarean delivery	1692 (36.8%)	74 (36.1%)			90 (32.7%)		
Vacuum extraction	226 (4.9%)	13 (6.3%)			9 (3.3%)		
Forceps extraction	16 (0.3%)	2 (1.0%)			2 (0.7%)		

*IHA* Inhibin-A, *FGR* fetal growth restriction;

**Table 3:** Crude and adjusted odd ratios for the risk of preterm birth and preeclampsia derived from logistic regression analysis

Outcomes	Preterm Birth (< 37 wk)				Preeclampsia			
Potential risk factors	Crude P-value	Crude Odd Ratios (95%CI)	Adjusted P-value	Adjusted Odd Ratios (95%CI)	Crude P-value	Crude Odd Ratios (95%CI)	Adjusted P-value	Adjusted Odd Ratios (95%CI)
Maternal age	0.049	0.99 (0.99–1.00)	0.030	0.99 (0.99–1.00)	0.984	1.00 (0.99–1.01)	0.030	0.99 (0.99–1.01)
Body weight	0.475	1.00 (0.99–1.01)	0.904	1.00 (0.97–1.03)	0.110	0.99 (0.97–1.00)	0.904	1.02 (0.98–1.06)
Body mass index	0.609	1.01 (0.98–1.03)	0.685	1.01 (0.95–1.08)	0.052	0.96 (0.92–1.00)	0.685	0.91 (0.81–1.01)
Socioeconomic status (high / low)	0.094	0.83 (0.66–1.03)	0.068	0.80 (0.63–1.02)	0.589	1.12 (0.75–1.66)	0.068	1.05 (0.70–1.57)
Inhibin-A levels (high / normal)	0.045	1.55 (1.01–2.39)	0.475	1.19 (0.74–1.90)	< 0.001	3.81 (2.17–6.68)	0.475	3.77 (2.15–6.63)
Parity (nulliparous / multiparous)	0.744	0.96 (0.79–1.18)	0.290	0.89 (0.72–1.11)	0.001	1.86 (1.28–2.71)	0.290	2.07 (1.40–3.06)
Preeclampsia (yes / no)	< 0.001	15.71 (10.70–23.07)	< 0.001	16.17 (10.94–23.92)	–	–	< 0.001	–

**Fig. 2:**
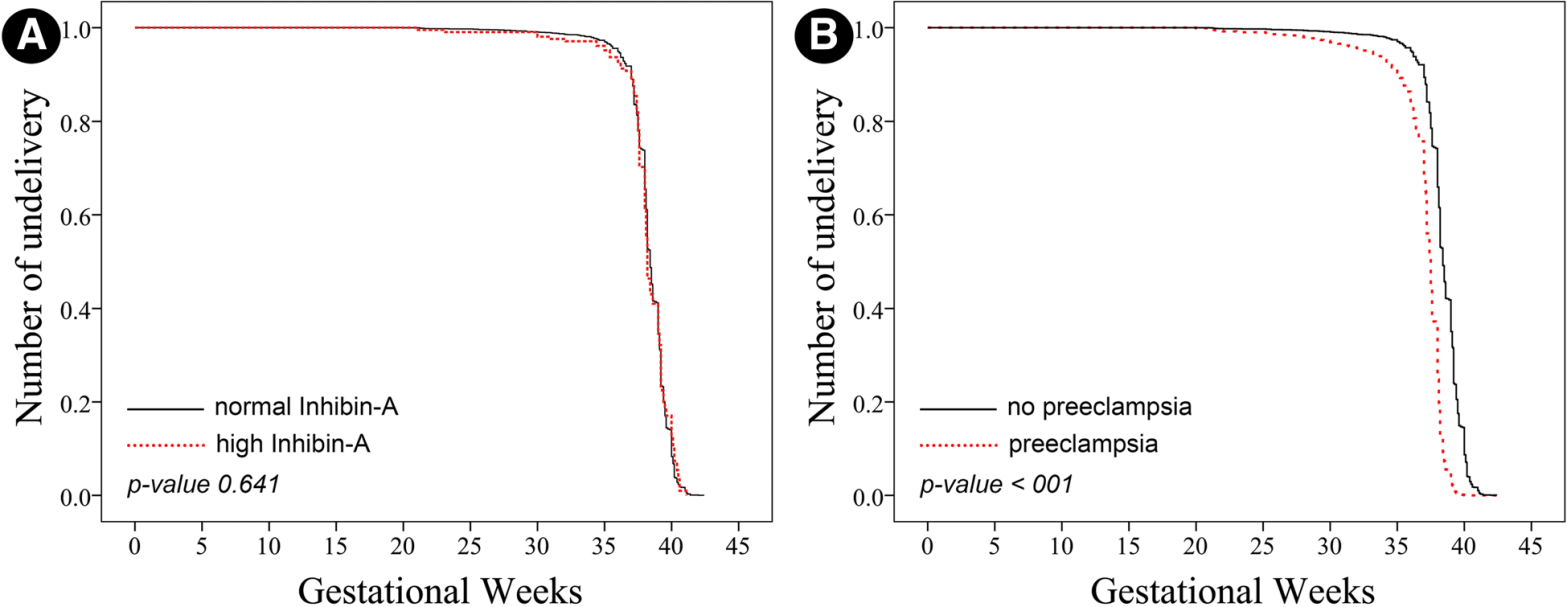
Kaplan-Meier curves of gestational age derived from Cox regression analysis show comparisons between the women with normal and high inhibin-A group (**a**), and the women with and without preeclampsia (**b**)

Regarding the comparison of Kaplan Meier curve of gestational age at delivery between the women with high and normal inhibin-A levels, Cox regression analysis showed that after adjusting for potential confounding factors, gestational age at delivery was not significantly different (*p*-value 0.641; odd ratio 1.02; 95% CI: 0.89–1.17), as presented in Fig. 2a, whereas gestational age was significantly different between the women with and without preeclampsia (*p*-value < 0.001; odd ratio 3.34; 95% CI: 2.77–4.04), as presented in Fig. 2b.

### Comparison of the adverse obstetric outcomes between the group of low and normal inhibin-a levels

In the comparison of pregnancy outcomes, including preeclampsia, preterm delivery, fetal growth restriction, low birth weight, low APGAR scores, antepartum hemorrhage, postpartum hemorrhage and route of delivery, the rates of all adverse outcomes in the group of low levels of serum inhibin-A were not significantly different from those in the normal inhibin-A group, as presented in Table 2**.**

## Discussion

As a population-based study, our results can reflect the outcomes in real practice among the general population, including all socioeconomic classes. An important finding was that elevated inhibin-A levels in the second trimester significantly increased the risk of preeclampsia, which contributed to a higher risk of overall and indicated preterm birth and fetal growth restriction. However high inhibin-A levels were not significantly associated with spontaneous preterm birth. Additionally, high levels of inhibin-A also significantly increased the risk of low-birth weight fetuses, which was likely to be a consequence of fetal growth restriction or preeclampsia rather than spontaneous preterm birth. Notably, low inhibin-A levels were not associated with any common adverse pregnancy outcomes.

In most previous reports, the associations between maternal serum markers and adverse pregnancy outcomes have been studied in western women but rarely studied in different geographical areas. However, our findings indicate that such associations were also reproducible among Thai population, though ethnicity and maternal body size significantly influence the levels of all maternal serum biomarkers.

With respect to literature, our findings are consistent with most studies in terms of overall adverse pregnancy outcomes, specifically preterm birth, fetal growth restriction and preeclampsia [9–11, 13]. Nevertheless, whereas most previous studies demonstrated a significant association between elevated inhibin-A levels and the risk of preterm birth [9, 14–16], they did not perform subgroup analysis for indicated and spontaneous preterm birth. In contrast, we determined whether abnormal inhibin-A levels increased the risk of indicated or spontaneous preterm birth or both. This is an important issue because prevention of preeclampsia with indicated preterm birth and prevention of spontaneous preterm birth are clinically different. In this study, logistic regression analysis of the preterm birth subgroup showed that the rate of preterm birth was significantly associated with preeclampsia and maternal age but not with inhibin-A levels. Therefore, we hypothesize that the high prevalence of preterm birth in cases of high inhibin-A levels shown in previous studies [10, 15] might have been a consequence of preeclampsia or fetal growth restriction rather than spontaneous preterm birth. This new insight may be clinically important because the measures of preterm birth prevention are different, depending on the pathogenesis of preterm birth. For example, spontaneous preterm birth without underlying causes can be successfully prevented by progesterone prophylaxis for quiescence of the uterus [3], while preterm birth associated with preeclampsia may benefit from aspirin prophylaxis to improve microcirculation and anti-platelet aggregation [4]. Our findings together with other previous studies indicate that elevated inhibin-A in the second trimester might be reflective of subtle abnormal development of the placenta in early gestation which could cause obvious clinical manifestations (fetal growth restriction and preeclampsia) in late pregnancy. Thus high inhibin-A in the second trimester may be considered as one of the risk factor of abnormal placentation, and aspirin may theoretically be helpful, though the effectiveness of this prophylaxis must be confirmed by further studies.

The association between elevated inhibin-A and adverse pregnancy outcomes is unclear. However, Fitzgerald B et al. [17] demonstrated that elevation of inhibin-A levels in the second trimester might be caused by premature accelerated differentiation of the villous cytotrophoblast, resulting in marked alterations in the syncytiotrophoblast morphology and concurrent villous cytotrophoblast depletion. As a result, the subsequent pathology in the syncytiotrophoblast could render the pregnancy at risk of fetal growth restriction and preeclampsia.

The weaknesses of this study are as follows: 1) The sample size was relatively small for comparisons of some rare secondary outcomes such as fetal death. 2) There were a significant number of women with unavailable data or loss to follow-up. 3) Since all participants resided in the northern part of Thailand, the outcomes may not be generalized to other geographical areas. 4) Analysis of the effects of combination of inhibin-A with other serum biomarkers on pregnancy outcomes was not performed. The strengths of this study are as follows: 1) The obstetric outcomes were followed up by the research group and prospectively recorded to the database. 2) Most of the known possible confounding factors were excluded prior to analysis, such as fetal anomaly, chromosome abnormalities, maternal medical diseases, etc. 3) All lab tests were carried out by the same experts, using the same lab machine in the same settings. 4) The network of hospitals included both urban and rural people, more reflective of all our population than tertiary referral centers or hospitals in the private sector.

## Conclusion

Elevation of maternal serum inhibin-A in the second trimester is significantly associated with abnormal placentation, which increases the risk of preeclampsia and fetal growth restriction with a consequence of indicated preterm birth but not significantly associated with spontaneous preterm birth. In contrast, low inhibin-A levels are not associated with any common adverse pregnancy outcome.

## Abbreviations

AFP: Alpha-fetoprotein
FGR: Feta growth restriction
hCG: Beta-human gonadotropin
MoM: Multiple of medians
uE3: Unconjugated estriol
